# Association between parental separation and addictions in adolescents: results of a National Lebanese Study

**DOI:** 10.1186/s12889-020-09108-3

**Published:** 2020-06-19

**Authors:** Nour Jabbour, Vanessa Abi Rached, Chadia Haddad, Pascale Salameh, Hala Sacre, Rabih Hallit, Michel Soufia, Sahar Obeid, Souheil Hallit

**Affiliations:** 1 Faculty of Medicine and Medical Sciences, Holy Spirit University of Kaslik (USEK), Jounieh, Lebanon; 2 Research and Psychology Departments, Psychiatric Hospital of the Cross, Jal Eddib, Lebanon; 3 Université de Limoges, UMR 1094, Neuroépidémiologie Tropicale, Institut d’Epidémiologie et de Neurologie Tropicale, GEIST, 87000 Limoges, France; 4 INSPECT-LB. Institut National de Santé Publique, Épidémiologie Clinique et Toxicologie – Liban, Beirut, Lebanon; 5 Faculty of Medicine, Lebanese University, Hadat, Lebanon; 6 Faculty of Pharmacy, Lebanese University, Hadat, Lebanon; 7 Faculty of Arts and Sciences, Holy Spirit University of Kaslik (USEK), Jounieh, Lebanon

**Keywords:** Divorce, Alcohol use disorder, Cigarette dependence, Waterpipe dependence, Addiction, Adolescents

## Abstract

**Background:**

Since divorce rates are on the rise in Lebanon (an increase of 101% between 2006 and 2017) and since previous international studies have shown a relationship between divorced parents and adolescents’ addiction to smoking, alcohol, and the internet, assessing the background of the Lebanese situation was deemed necessary. The study objective was to investigate the association between the divorce of parents and smoking, alcohol, and internet addiction among a representative sample of Lebanese adolescents.

**Methods:**

This study was a cross-sectional, conducted between January and May 2019 using a proportionate random sample of schools from all Lebanese Mohafazat. Out of 2000 questionnaires distributed; 1810 (90.5%) were completed and collected back.

**Results:**

The mean age was 15.42 ± 1.14 years, with 53.3% females and 74.1% smokers. In addition, 11.9% [95% CI 0.104–0.134] of the adolescents had separated/divorced parents. Divorce in parents was significantly associated with higher alcohol use disorder (Beta = 8.035), higher cigarette dependence (Beta = 2.767) and a higher waterpipe dependence (Beta = 5.263) in adolescents. However, divorce in parents was not associated with internet addiction in adolescents.

**Conclusion:**

Parental divorce is correlated to higher alcohol and smoking, but not internet addiction among adolescents. Children whose parents are divorced should be subject to continuous follow-up by their parents and by a psychiatrist/psychologist in order not to develop an addiction that could potentially harm them.

## Background

Adolescence is the transitional phase between late childhood and the beginning of adulthood [1], during which adolescents undergo rapid changes compared to other developmental periods. Psychologically, identity is formed, including self-exploration for interpersonal goals, i.e., the need for social friendship and the determination for an autonomous, organized personality [2]. At the cognitive level, adolescents develop general thinking, allowing for a hardly noticeable formulation of a multifaceted personality and a growing concern in appreciating and accepting one’s self compared to others [3]. During this complex phase [4], adolescents face biological and experiential changes and chart their lifestyles, which will have long-term impacts on all facets of their growth, including health. Risky behaviors occur naturally during adolescence and can extend into adulthood; cigarette and waterpipe smoking and alcohol use are among the main risky behaviors initiated during this period and might become addictions [5]. The American Society of Addiction Medicine (ASAM) defines addiction as “*a primary chronic disease of brain reward, motivation, memory, and related circuitry*” [6]. To further explain addictions, the Diagnostic and Statistical Manual of Mental Disorders- 5th edition (DSM-5) (APA, 2013) [7] expanded the chapter on substance-related disorders to include not just the types of psychoactive substances but also addictive behaviors, particularly the internet addiction [8].

Moreover, according to Problem Behavior Theory [9], first proposed by Jessor, the family structure (divorce, communication between parents and child) predicts externalizing problems (smoking, alcohol drinking).

Previous studies have shown that divorce induced a higher risk of alcohol onset among students rather than parental drinking [10, 11]. Also, adolescents from non-intact families (defined as children who did not live with both biological parents at the end of childhood due to parental separation or single motherhood at birth) started alcohol drinking at a younger age and reported frequent and heavy drinking, and drunkenness compared to those from intact families [10]. The same study showed that 32% of adolescents as young as 11 to 13 years of age, who experienced parental separation/divorce, reported having drunk a full alcoholic beverage [10]. In Lebanon, alcohol use during adolescence is high due to the ease of purchase (no age restriction, cheap price < 2$), and the lack of strict laws against alcohol [12].

The prevalence of cigarette smoking among adolescents is on the rise worldwide and varies between 10.4% [13] and 51% [14], mostly occurring in developing countries [15]; Lebanon ranks first in the Middle East and among Arab women, with 43% of men and 28% of women in Lebanon being smokers [15]. Waterpipe smoking prevalence is also high in Lebanon, especially in youth, with 35% of teens aged between 13 and 15 having used it; among those, 47% smoked less than once weekly, 38% weekly but not daily, and 16% daily [16]. A study had demonstrated that divorce increased the risk of initiating drinking and smoking [17]; however, to our knowledge, no studies have yet evaluated the association between divorce and waterpipe addiction during adolescence.

The number of Internet users is growing exponentially worldwide, exceeding 2.5 billion active users, particularly adolescents [18–20]. Teenagers with internet addiction are more likely to have divorced parents and emotional problems, with most of them being males [21]. Many factors, including but not limited to the environment, emotional immaturity, and the inability to cope with negative emotions, are underlying reasons for internet addiction among adolescents, emphasizing the possible relationship between divorce and internet addiction in this age group [22]. A study covering determinants of internet addiction during adolescence shows that 7% of adolescent internet addicts have dysfunctional and/or problematic family relationships [23].

Not every adolescent is at equal risk of developing an addiction. Many studies [23, 24] have investigated various aspects of addiction among adolescents and determined the reason behind it and why some adolescents are more prone to addiction than others. Susceptibility differs because the vulnerability of people varies according to different genetic, environmental, and developmental factors, such as the Western and Eastern culture [25].

Since divorce rates are steadily growing in Lebanon (an increase of 101% between 2006 and 2017) [26, 27], and since previous international studies [11, 28–31] have shown a relationship between divorced parents and adolescents’ addiction to smoking, alcohol, and the internet, assessing the background of the Lebanese situation was deemed necessary. The practical implication of our study relates to evaluating the possible influence of previous or recent parental divorce on adolescent addiction to warn the parents about the importance of secure attachment. Therefore, the objective of our study was to examine the association between the divorce of parents and smoking, alcohol, and internet addiction among a representative sample of Lebanese adolescents.

## Methods

### Participants

A cross-sectional study was conducted between January and May 2019. It enrolled students aged between 14 and 17, from a random sample of private schools from all Lebanese Mohafazat/districts (Beirut, Mount Lebanon, North, South, and Beqaa). A total of 18 private schools was contacted, two refused to participate. Those who accepted were distributed as follows: 4 in Beirut, 2 in South Lebanon, 6 in Mount Lebanon, 2 in North Lebanon, and 2 in Beqaa. All students in this age bracket were eligible. Excluded were those who refused to participate. No compensation was offered in exchange for participation. Out of 2000 questionnaires distributed, 1810 (90.5%) were completed and collected back. The methodology used in this study is similar to that of previous publications [32–34].

### Questionnaire

The questionnaire used was anonymous, in Arabic (the native language in Lebanon), and required approximately 60 min to complete. Students filled it in the classrooms to avoid their parents’ influence while answering the questions. At the end of the process, the completed questionnaires were collected back and sent for data entry.

The first part assessed the sociodemographic details of the participants (i.e., age, gender, smoking status, parents’ status). Other features were also recorded: the heights and weights of participants to calculate the Body Mass Index (BMI) (kg/m^2^); the number of persons living in the house and the number of rooms in the house, excluding the bathroom and the kitchen, to calculate the household crowding index [35]; the intensity, duration, and frequency of daily activity as reported by the students to compute the Total Physical Activity Index [36]. The intensity was evaluated as sustained heavy breathing and perspiration (5 points), intermittent heavy breathing and perspiration (tennis, racquetball) (4 points), moderate-heavy (recreational sports, cycling) (3 points), moderate (volleyball) (2 points), and light (fishing, walking) (1 point). As for the duration, 4 points were attributed for exercising for over 30 min daily, 3 points for 20–30 min daily, 2 points for 10–20 min daily, and 1 point for a daily exercise of under 10 min daily. The frequency of exercising was divided into daily/almost daily (5 points), 3–5 times/week (4 points), 1–2 times/week (3 points), few times a month (2 points), and less than once a month (1 point).

The second part of the questionnaire included the following scales:

#### Internet addiction test (IAT)

It consisted of 20 items, with Likert type responses varying between 0 (does not apply/never) and 5 (always applies). The total score ranged between 20 and 100, with higher scores defining higher internet addiction (Cronbach’s alpha = 0.925).

#### The alcohol use disorders identification test (AUDIT)

The 10-item self-reported version of the AUDIT, validated in Lebanon [33], was used in this study to assess alcohol use, drinking patterns, and alcohol-related issues [37]. Hazardous Alcohol Disorder (HAD) is considered when the patients score 8 or more (Cronbach’s alpha = 0.960).

#### Lebanon Waterpipe dependence Scale-11 (LWDS-11)

The LWDS-11 test was used to assess waterpipe dependence [38]. It includes 11 items measured on a 4-point Likert scale ranging from 0 to 3. The total score is calculated by summing all responses, with higher scores indicating higher waterpipe addiction (Cronbach’s alpha = 0.888).

#### Fagerström test for nicotine dependence (FTND)

The FTND includes six items; dichotomous questions are scored 0 and 1, and multiple-choice items are graded from 0 to 3. All answers are summed to yield a total score of 0–10. The higher the total Fagerström score, the more intense the physical dependence on nicotine [39] (Cronbach’s alpha = 0.825).

### Translation procedure

The forward translation was done by a health professional, whose native language is Arabic and fluent in English. A backward translation was then performed by a native English speaker translator, fluent in Arabic, and unfamiliar with the concepts of the scales. The back-translated English questionnaire was subsequently compared to the original English one, aiming to discern discrepancies and to solve any inconsistencies between the two versions.

### Statistical analysis

SPSS software version 23 was used to conduct data analysis. Cronbach’s alpha values were recorded for the scales’ reliability analysis. Counts and percentages, and means and standard deviations, were calculated for categorical and continuous variables, respectively.

A multivariate analysis of covariance (MANCOVA) was carried out to compare multiple scales scores (taken as dependent variables) and the parents’ status (living together vs. separated), after adjusting over potential confounding variables: age, gender, house crowding index, and physical activity score. A value of *p* < 0.05 was considered significant.

## Results

Table 1 summarizes the sociodemographic characteristics of the participants. The mean age was 15.42 ± 1.14 years, with 53.3% females, 74.1% nonsmokers, and 11.9% [95% CI 0.104–0.134] having separated/divorced parents.

**Table 1:** Sociodemographic characteristics of the sample population

	**Frequency (%)**
**Gender**	
Male	844 (46.7%)
Female	963 (53.3%)
**Parents status**	
Living together	1581 (88.1%)
Separate	213 (11.9%)
**Smoking status**	
Yes	468 (25.9%)
No	1342 (74.1%)
	**Mean ± SD**
**Age (years)**	15.42 ± 1.14
**Body Mass Index (kg/m2)**	21.95 ± 4.21
**Household crowding index**	1.01 ± 0.64

**Fig. 1:**
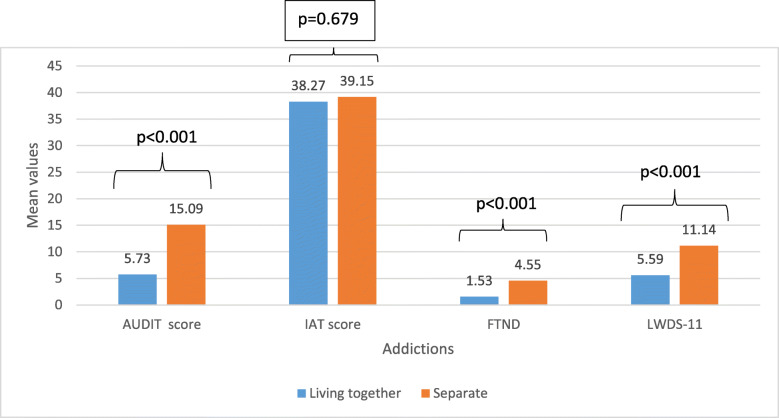
Mean values of the addiction scales according to parent status adjusted for age, gender, house crowding index and physical activity

Figure 1 shows the mean values of the addiction scales adjusted for age, gender, house crowding index, and physical activity, according to parent status. After adjusting for all covariates, a significantly higher mean of AUDIT (15.09), FTND (4.55), and LWDS-11 (11.14) scores were found in adolescents whose parents are separated as compared to those whose parents are living together. No significant difference was found for the IAT total score.

### Multivariate analysis

The MANCOVA analysis was performed taking the scales as the dependent variables and the groups of parents’ status (living together vs. separate) as the independent variable, after adjusting for the covariates (age, gender, house crowding index, and physical activity score).

Considering the AUDIT score as the dependent variable, adolescents whose parents are separated compared to living together (Beta = 8.035) and higher physical activity index (Beta = 0.06) were significantly associated with higher AUDIT scores.

Taking the IAT score as the dependent variable, being a female (Beta = 2.697), older age (Beta = 0.991), and higher physical activity index (Beta = 0.234) were significantly associated with higher IAT scores.

Taking the FTND score as the dependent variable, adolescents whose parents are separated compared to living together (Beta = 2.767) were significantly associated with higher FTND scores. Older age (Beta = − 0.333) and higher house crowding index (Beta = − 0.357) were significantly associated with lower FTND scores.

Taking the LWDS-11 score as the dependent variable, adolescents whose parents are separated compared to living together (Beta = 5.263) were significantly associated with higher LWDS-11 scores. Older age (Beta = − 1.293) and higher house crowding index (Beta = − 1.098) were significantly associated with lower LWDS-11 scores (Table 2).

**Table 2:** Multivariate analysis of covariance (MANCOVA)

	**Beta**	***p*-value**	**95% Confidence Interval**		**Partial Eta Squared**
			**Lower Bound**	**Upper Bound**	
**AUDIT total score**					
Age	0.332	0.064	−0.019	.683	0.001
Gender (females vs males^a^)	0.295	0.493	−0.548	1.137	0.001
Parents status (separated vs living together^a^)	8.035	**< 0.001**	6.840	9.230	0.10
House crowding index	−0.161	0.624	−0.805	0.483	0.001
Physical activity score	0.060	**< 0.001**	0.032	0.089	0.003
**IAT total score**					
Age	0.991	**0.017**	0.178	1.803	0.006
Gender (females vs males*)	1.695	0.070	−0.136	3.526	0.002
Parents status (separated vs living together*)	−0.584	0.679	−3.348	2.181	0.001
Physical activity score	0.234	**< 0.001**	0.168	0.301	0.03
House crowding index	1.141	0.084	−0.154	2.436	0.001
**FTND**					
Age	−0.333	**< 0.001**	−0.462	− 0.203	0.02
Gender (females vs males*)	−0.097	0.540	−0.409	0.214	0.001
Parents status (separated vs living together*)	2.767	**< 0.001**	2.325	3.209	0.09
House crowding index	−0.357	**0.003**	−0.595	− 0.119	0.005
Physical activity score	0.005	0.354	−0.006	0.016	0.001
**LWDS-11**					
Age	−1.293	**< 0.001**	−1.687	−0.899	0.03
Gender (females vs males*)	−0.966	**0.029**	1.834	−0.099	0.003
Parents status (separated vs living together*)	5.263	**< 0.001**	3.923	6.604	0.044
House crowding index	−1.098	**0.003**	− 1.820	−0.376	0.005
Physical activity score	0.016	0.320	−0.016	0.048	0.001

Note: In the global model, the independent variable is Parents status (living together* vs. separate). Covariates are: age, gender, house crowding index and physical activity score

*Reference group; numbers in bold indicate significant p-values

The MANCOVA analysis, taking all the scales as dependent variables and adjusted for the covariates (age, gender, house crowding index, physical activity score, and Mouhafaza/district), can be found in Supplementary Table 1. Living in Mount Lebanon was significantly associated with higher cigarette and waterpipe dependence compared to living in Beirut while living in the North, South, and Beqaa was significantly associated with lower alcohol use disorder and cigarette dependence compared to living in Beirut.

## Discussion

This cross-sectional national study, conducted on a group of adolescents across all districts in Lebanon, aimed to assess the potential risk factors for addiction among adolescents, particularly the effect of parental status on adolescents’ risky behavior. The results of the study showed that adolescents whose parents are divorced or separated were more likely to have hazardous alcohol disorder (HAD), greater dependence on cigarette and waterpipe, but not higher internet addiction.

Parental divorce was significantly associated with possible HAD among adolescents, in agreement with the findings of a previous study [30]. Indeed, parental divorce is among the most frequently recognized adverse events encountered during childhood, which has been associated with higher odds of alcohol consumption and HAD in adolescence and early adulthood. Divorce is expected to increase possible HAD as it reduces the supervision of adolescents, decreases parental effectiveness, and increases access to alcohol, as parents are busy solving their problems. In that case, alcohol use in adolescents may be due to more propensity to seek out new and potentially dangerous situations and meet emotional needs, especially that the parents are at first busy trying to solve their problems. Another possible reason would be that parental divorce may lead to higher affiliation with substance-using peers, thereby increasing alcohol consumption among adolescents [28, 40]. Hence, the absence of monitoring by separated parents leads to outings with unusual peers, increased access to alcohol, and eventually earlier age of drinking initiation [10]. Parental divorce may have less influence on adolescents’ risky behaviors once they become more independent and less reliant on parental support [10].

Furthermore, our results showed that parental divorce was not associated with higher internet addiction among adolescents, contrary to previous findings [29, 31]. Mann’s notion of availability as a law of addiction [41] suggests that greater internet availability may foster greater engagement in online activities. It is noteworthy that internet accessibility is not homogenous on the Lebanese territory and that many adolescents do not have full-time access to the internet or electronic devices. Another reason might be the emergence of parental control software that blacklist certain types of websites, apply time limits, and schedule internet access [42].

Finally, consistent with previous studies [11, 30], our results showed that adolescents whose parents are separated had higher odds of smoking dependence. The period of parental conflict is a significant stressor for children, which can produce an aversive home environment that may be responsible for the negative correlation with health outcomes. Extensive research has attempted to identify some of the main risk factors for smoking initiation; it appears that psychological distress, such as a depressed mood, and high levels of rebelliousness may be triggers for nicotine addiction [43, 44]. Another explanation might be that, at this age, without rigorous parental supervision, adolescents are unable to grasp the long-term risks and consequences of smoking and often feel like it is their “self-medication” rather than a potential risk factor for subsequent diseases [45]. Moreover, the uptake of a new, potentially harmful habit could be a way for adolescents to grab their parent’s attention when the parent-child bond is weakened. It is noteworthy that our study was conducted in a Middle-Eastern country where waterpipe smoking is highly prevalent and part of the cultural, traditional, and social heritage [11, 46]. It is also perceived as a means of socialization, entertainment, and relaxation, thus popular among young people [47].

### Sociodemographic characteristics

Female gender was significantly associated with lower waterpipe dependence, in line with other studies [48, 49]. The explanation could be that the prevalent culture in Lebanon accepts more male rather than female smokers [50]. Further, older age was significantly associated with lower smoking scores, in line with one previous study [51] but contradictory with others [48, 52]. It is hypothesized that these older adolescents may have a higher awareness of the health consequences of smoking than younger adolescents [51]. In addition, higher house crowding index was associated with lower smoking addiction in adolescents, in line with previous research [53, 54]. This negative association can be hypothesized by the fact that a crowded house does not give to adolescent’s freedom or the time to smoke alone, especially if their roommate is annoyed by passive smoking [32].

Higher physical activity index was significantly associated with higher AUDIT scores, in line with previous studies [55, 56]. Although the factors and direction of the association between physical activity and alcohol addiction are unclear, previous research speculates that both behaviors may have a mutual influence [57]. Active people tend to be exposed to a more frequent social interaction, which in turn could lead to higher alcohol consumption; thus, alcohol consumers could practice more physical activity to compensate for drinking alcohol [58].

Further, older age was significantly associated with higher internet addiction, in line with previous findings [59] showing that with age, adolescents have a higher level of independence; their leisure time and social activities are less controlled by their parents.

### Clinical implications

The high rates of addiction (cigarette and waterpipe smoking and alcohol drinking) among adolescents whose parents are divorced, revealed by our results, highlight the urgent need for health managers and policymakers to develop evidence-based interventions to reduce the risk and consequences of divorce. Increasing parental education in such situations is needed to improve parents’ communication proficiency, a necessary component in reducing the adolescent’s emotional suffering and loneliness, and achieve healthy familial interactions.

### Limitations

This study has a few limitations. Its cross-sectional design does not infer causality (the temporal occurrence of the events is unknown). It did not assess the duration for which adolescents were exposed to parental separation nor that of each addiction. Also, for quitters, no enough data was available regarding the time they stopped smoking; therefore, it is not known whether quitting was done before or after parental divorce. Some of the questions may not have been correctly understood, thus leading to information bias. Also, the study did not take into consideration adolescents who do not attend schools. Finally, the schools’ selection process might have caused a selection bias.

## Conclusion

Parental divorce is correlated to higher alcohol and smoking, but not internet addiction among adolescents. Divorced parents should always follow-up on their children to prevent them from developing behaviors that could potentially harm them.

## Supplementary information

**Additional file 1: Table S1.** Multivariate analysis of covariance (MANCOVA). Note: In the global model, the independent variable is Parents status (living together* vs. separate). Covariates are: age, gender, mouhafaza, house crowding index and physical activity score. *Reference group.

## Data Availability

All data generated or analyzed during this study are not publicly available to maintain the privacy of the individuals’ identities. The dataset supporting the conclusions is available upon request to the corresponding author.

## Abbreviations

ASAM: American Society of Addiction Medicine
DSM-5: The Diagnostic and Statistical Manual of Mental Disorders- 5th edition
BMI: Body Mass Index
IAT: Internet Addiction Test
AUDIT: Alcohol Use Disorders Identification Test
HAD: Hazardous Alcohol Disorder
LWDS-11: Lebanon Waterpipe Dependence Scale-11
FTND: Fagerström test for nicotine dependence
MANCOVA: multivariate analysis of covariance

## References

[1] Casey B, Duhoux S, Cohen MM (2010). Adolescence: what do transmission, transition, and translation have to do with it?. Neuron..

[2] La Guardia JG (2009). Developing who I am: a self-determination theory approach to the establishment of healthy identities. Educ Psychol.

[3] Trucco EM, Wright AG, Colder CR (2014). Stability and change of social goals in adolescence. J Pers.

[4] Richter LM (2006). Studying adolescence. Science..

[5] Mak KK, Ho SY, Thomas GN, Schooling CM, McGhee SM, Lam TH (2010). Family structure, parent-child conversation time and substance use among Chinese adolescents. BMC Public Health.

[6] Smith DE (2012). The process addictions and the new ASAM definition of addiction. J Psychoactive Drugs.

[7] American Psychiatric Association, DSM-5 Task Force. Diagnostic and statistical manual of mental disorders: DSM-5™ (5th ed.). American Psychiatric Publishing, Inc. 2013. 10.1176/appi.books.9780890425596.

[8] Grant JE, Chamberlain SR (2016). Expanding the definition of addiction: DSM-5 vs. ICD-11. CNS spectrums.

[9] Jessor R, Jessor SL (1977). Problem behavior and psychosocial development: a longitudinal study of youth.

[10] Jackson KM, Rogers ML, Sartor CE (2016). Parental divorce and initiation of alcohol use in early adolescence. Psychol Addict Behav.

[11] Soares ALG, Gonçalves H, Matijasevich A (2017). Parental separation and Cardiometabolic risk factors in late adolescence: a cross-cohort comparison. Am J Epidemiol.

[12] Yassin N, Afifi R, Singh N, Saad R, Ghandour L (2018). “There is zero regulation on the selling of alcohol”: the voice of the youth on the context and determinants of alcohol drinking in Lebanon. Qual Health Res.

[13] Huang HW, Lu CC, Yang YH, Huang CL (2014). Smoking behaviours of adolescents, influenced by smoking of teachers, family and friends. Int Nurs Rev.

[14] Hallal ALC, Gotlieb SLD, Almeida LMd, Casado L. Prevalence and risk factors associated with smoking among school children, southern Brazil. Revista de Saude Publica 2009;43(5):779–788. 10.1590/s0034-89102009005000056 19768233

[15] Salti N, Brouwer E, Verguet S (2016). The health, financial and distributional consequences of increases in the tobacco excise tax among smokers in Lebanon. Soc Sci Med.

[16] Bahelah R, DiFranza JR, Ward KD (2017). Waterpipe smoking patterns and symptoms of nicotine dependence: the Waterpipe dependence in Lebanese youth study. Addict Behav.

[17] Hsu Y-T, Kawachi I (2019). Timing of family adversity during adolescence and its impact on alcohol and tobacco initiation: a longitudinal study among Taiwanese adolescents. Child Psychiatry Hum Dev.

[18] Internet World Stats. Usage and Population Statistics. 2019. Available from: https://internetworldstats.com/stats.htm. [Accessed 30 Sept 2019].

[19] Social Networking Reaches Nearly One in Four Around the World. 2013. Available at: https://www.emarketer.com/Article/Social-Networking-Reaches-Nearly-One-Four-Around-World/1009976. Accessed 30 Sept 2019.

[20] Obeid S, Saade S, Haddad C, et al. Internet Addiction Among Lebanese Adolescents: The Role of Self-Esteem, Anger, Depression, Anxiety, Social Anxiety and Fear, Impulsivity, and Aggression-A Cross-Sectional Study. J Nerv Ment Dis. 2019;207(10):838–46. 10.1097/NMD.0000000000001034. 10.1097/NMD.0000000000001034 31503174

[21] Yen JY, Yen CF, Chen CC, Chen SH, Ko CH (2007). Family factors of internet addiction and substance use experience in Taiwanese adolescents. CyberPsychol Behav.

[22] Telef BB (2016). Investigating the relationship among internet addiction, positive and negative affects, and life satisfaction in Turkish adolescents. Int J Progress Educ.

[23] Tsitsika A, Critselis E, Louizou A (2011). Determinants of internet addiction among adolescents: a case-control study. Sci World J.

[24] Gamez-Guadix M, Calvete E, Orue I, Las HC (2015). Problematic internet use and problematic alcohol use from the cognitive-behavioral model: a longitudinal study among adolescents. Addict Behav.

[25] Ellis BJ, Boyce WT, Belsky J, Bakermans-Kranenburg MJ, Van IJzendoorn MH (2011). Differential susceptibility to the environment: an evolutionary–neurodevelopmental theory. Dev Psychopathol.

[26] Women Economic Empowerment Portal. Divorce rate in Lebanon doubles in 10 years. Available at: https://www.weeportal-lb.org/news/divorce-rate-lebanon-doubles-10-years. [Accessed 30 Sept 2019].

[27] ALJAZEERA WORLD. Divorce in Lebanon. Available at: https://www.aljazeera.com/programmes/aljazeeraworld/2017/01/divorce-lebanon-170117073031972.html. [Accessed 30 Sept 2019].

[28] National Institute on Alcohol Abuse and Alcoholism. Underage Drinking. Available at: https://pubs.niaaa.nih.gov/publications/AA67/AA67.htm. [Accessed 1 Oct 2019].

[29] Chung TW, Sum SM, Chan MW (2019). Adolescent internet addiction in Hong Kong: prevalence, psychosocial correlates, and prevention. J Adolesc Health.

[30] Doku DT, Acacio-Claro PJ, Koivusilta L, Rimpelä A. Social determinants of adolescent smoking over three generations. Scand J Public Health. 2019;1403494819839854. 10.1177/1403494819839854 30973093

[31] Wu CST, Wong HT, Yu KF (2016). Parenting approaches, family functionality, and internet addiction among Hong Kong adolescents. BMC Pediatr.

[32] Nakhoul L, Obeid S, Sacre H (2020). Attachment style and addictions (alcohol, cigarette, waterpipe and internet) among Lebanese adolescents: a national study. BMC Psychol.

[33] Hallit J, Salameh P, Haddad C (2020). Validation of the AUDIT scale and factors associated with alcohol use disorder in adolescents: results of a National Lebanese Study. BMC Pediatr.

[34] Chahine, M., Salameh, P., Haddad, C. et al. Suicidal ideation among Lebanese adolescents: scale validation, prevalence and correlates. BMC Psychiatry 20, 304 (2020). 10.1186/s12888-020-02726-6. 10.1186/s12888-020-02726-6 PMC7296775 32539735

[35] Melki I, Beydoun H, Khogali M, Tamim H, Yunis K (2004). Household crowding index: a correlate of socioeconomic status and inter-pregnancy spacing in an urban setting. J Epidemiol Community Health.

[36] Weary-Smith KA. Validation of the physical activity index (PAI) as a measure of total activity load and total kilocalorie expenditure during submaximal treadmill walking, University of Pittsburgh; 2007. 10.1123/jpah.9.6.757 21952161

[37] Bohn MJ, Babor TF, Kranzler HR (1995). The alcohol use disorders identification test (AUDIT): validation of a screening instrument for use in medical settings. J Stud Alcohol.

[38] Salameh P, Waked M, Aoun Z (2008). Waterpipe smoking: construction and validation of the Lebanon Waterpipe dependence scale (LWDS-11). Nicotine Tob Res.

[39] Heatherton TF, Kozlowski LT, Frecker RC, FAGERSTROM KO (1991). The Fagerström test for nicotine dependence: a revision of the Fagerstrom tolerance questionnaire. Br J Addict.

[40] Kuntsche EN, Kuendig H (2006). What is worse? A hierarchy of family-related risk factors predicting alcohol use in adolescence. Subst Use Misuse.

[41] MANN RE. Availability as a law of addiction. Addiction. 2005;100(7):924–5. 10.1111/j.1360-0443.2005.01146.x 15955003

[42] Comparitech. Technology and internet addiction: How to recognize it and recover from it. Available at: https://www.comparitech.com/internet-providers/technology-internet-addiction/. Accessed 30 Sept 2019.

[43] Savolainen I, Kaakinen M, Sirola A, Oksanen A (2018). Addictive behaviors and psychological distress among adolescents and emerging adults: a mediating role of peer group identification. Addict Behav Rep.

[44] Primack BA, Land SR, Fan J, Kim KH, Rosen D (2013). Associations of mental health problems with waterpipe tobacco and cigarette smoking among college students. Subst Use Misuse..

[45] Morrell HE, Song AV, Halpern-Felsher BL (2010). Predicting adolescent perceptions of the risks and benefits of cigarette smoking: a longitudinal investigation. Health Psychol.

[46] Hall FS, Der-Avakian A, Gould TJ, Markou A, Shoaib M, Young JW (2015). Negative affective states and cognitive impairments in nicotine dependence. Neurosci Biobehav Rev.

[47] Shuja S, Hussain A, Malik S, Rizwan T, Amin M, Choudhry Z (2018). Perceptions of health professional students regarding Waterpipe smoking and its effects on Oral health. J Ayub Med College Abbottabad.

[48] Lim KH, Lim HL, Teh CH (2017). Smoking among school-going adolescents in selected secondary schools in peninsular Malaysia-findings from the Malaysian adolescent health risk behaviour (MyaHRB) study. Tob Induc Dis.

[49] Backhaus I, D'Egidio V, Grassucci D, Gelardini M, Ardizzone C, La Torre G (2017). Link between perceived smoking behaviour at school and students smoking status: a large survey among Italian adolescents. Public Health.

[50] Kalaboka S, Piau J, King G, Moreau D, Choquet M, Annesi-Maesano I. Sex and gender differences in tobacco smoking among adolescents in French secondary schools. Monaldi Arch Chest Dis. 2008;69(3):142–51. 10.4081/monaldi.2008.393. 10.4081/monaldi.2008.393 19065850

[51] Atikah AN, Wee LH, Zakiah MN (2019). Factors associated with different smoking statuses among Malaysian adolescent smokers: a cross-sectional study. BMC Public Health.

[52] Reidpath DD, Davey TM, Kadirvelu A, Soyiri IN, Allotey P (2014). Does one cigarette make an adolescent smoker, and is it influenced by age and age of smoking initiation? Evidence of association from the U.S. youth risk behavior surveillance system (2011). Prev Med.

[53] Habibi M, Hosseini F, Darharaj M, Moghadamzadeh A, Radfar F, Ghaffari Y (2018). Attachment style, perceived loneliness, and psychological well-being in smoking and non-Smoking University students. J Psychol.

[54] Lin M-P, Ko H-C, Wu JY-W (2011). Prevalence and psychosocial risk factors associated with internet addiction in a nationally representative sample of college students in Taiwan. Cyberpsychol Behav Soc Netw.

[55] Dodge T, Clarke P (2018). Testing weight motives and guilt/shame as mediators of the relationship between alcohol use and physical activity. Addict Behav.

[56] Buchholz LJ, Crowther JH (2014). Women who use exercise as a compensatory behavior: how do they differ from those who do not?. Psychol Sport Exerc.

[57] Conroy DE, Pincus AL, Ram N, al’Absi M. Thirsting to understand the temporal dynamics of physical activity and alcohol use. BMJ Publishing Group Ltd and British Association of Sport and Exercise Medicine; 2018. 10.1136/bjsports-2017-097949 28733360

[58] Werneck AO, Oyeyemi AL, Szwarcwald CL, Silva DR (2019). Association between physical activity and alcohol consumption: Sociodemographic and behavioral patterns in Brazilian adults. J Public Health.

[59] Karacic S, Oreskovic S (2017). Internet addiction through the phase of adolescence: a questionnaire study. JMIR Mental Health.

