# Supplementary material for: Association between parental separation and addictions in adolescents: results of a National Lebanese Study
Source: BMC Public Health. 2020 Jun 19;20:965. doi: 10.1186/s12889-020-09108-3 (PMC7304209; doi:10.1186/s12889-020-09108-3)
Supplement: Supplementary file 1 — Additional file 1: Table S1. Multivariate analysis of covariance (MANCOVA). Note: In the global model, the independent variable is Parents status (living together* vs. separate). Covariates are: age, gender, mouhafaza, house crowding index and physical activity score. *Reference group. [file 12889_2020_9108_MOESM1_ESM.docx]

| **Supplementary Table 1: Multivariate analysis of covariance (MANCOVA)** | | | | |  |
| --- | --- | --- | --- | --- | --- |
|  | **Beta** | **p-value** | **95% Confidence Interval** | | **Partial Eta Squared** |
|  |  |  | **Lower Bound** | **Upper Bound** |  |
| **AUDIT total score** |  |  |  |  |  |
| Age | 0.124 | 0.479 | -0.219 | 0.467 | 0.001 |
| Gender (females vs males*) | 0.337 | 0.417 | -0.478 | 1.152 | 0.001 |
| Parents status (separated vs living together*) | 7.567 | <0.001 | 6.373 | 8.760 | 0.091 |
| House crowding index | 0.528 | 0134 | -0.162 | 1.218 | 0.001 |
| Physical activity score | 0.032 | 0.024 | 0.004 | 0.059 | 0.003 |
| Mount Lebanon (compared to Beirut) | -0.566 | 0.434 | -1.984 | 0.852 | 0.001 |
| North Lebanon (compared to Beirut) | -2.953 | <0.001 | -4.331 | -1.575 | 0.011 |
| South Lebanon (compared to Beirut) | -3.580 | <0.001 | -5.016 | -2.144 | 0.015 |
| Bekaa (compared to Beirut) | -3.694 | <0.001 | -5.040 | -2.347 | 0.018 |
| **IAT total score** |  |  |  |  |  |
| Age | 1.267 | 0.001 | 0.494 | 2.040 | 0.007 |
| Gender (females vs males*) | 1.687 | 0.072 | -0.150 | 3.524 | 0.002 |
| Parents status (separated vs living together*) | -0.207 | 0.880 | -2.897 | 2.482 | 0.001 |
| House crowding index | -0.258 | 0.745 | -1.813 | 1.298 | 0.001 |
| Physical activity score | 0.198 | <0.001 | 0.136 | 0.259 | 0.025 |
| Mount Lebanon (compared to Beirut) | -2.315 | 0.156 | -5.512 | 0.882 | 0.001 |
| North Lebanon (compared to Beirut) | 1.509 | 0.341 | -1.597 | 4.616 | 0.001 |
| South Lebanon (compared to Beirut) | -2.372 | 0.151 | -5.610 | 0.65 | 0.001 |
| Bekaa (compared to Beirut) | -2.141 | 0.167 | -5.175 | 0.894 | 0.001 |
| **FTND** |  |  |  |  |  |
| Age | -0.397 | <0.001 | -0.514 | -0.280 | 0.028 |
| Gender (females vs males*) | 0.007 | 0.962 | -0.272 | 0.285 | 0.001 |
| Parents status (separated vs living together*) | 2.483 | <0.001 | 2.076 | 2.891 | 0.085 |
| House crowding index | 0.086 | 0.476 | -0.150 | 0.321 | 0.001 |
| Physical activity score | 0.001 | 0.924 | -0.010 | 0.009 | 0.001 |
| Mount Lebanon (compared to Beirut) | 0.804 | 0.001 | 0.319 | 1.288 | 0.007 |
| North Lebanon (compared to Beirut) | -0.771 | 0.001 | -1.242 | -0.300 | 0.007 |
| South Lebanon (compared to Beirut) | -0.552 | 0.027 | -1.043 | -0.062 | 0.003 |
| Bekaa (compared to Beirut) | -1.341 | <0.001 | -1.801 | -0.881 | 0.021 |
| **LWDS-11** |  |  |  |  |  |
| Age | -1.296 | <0.001 | -1.659 | -0.932 | 0.031 |
| Gender (females vs males*) | -0.722 | 0.101 | -1.587 | 0.142 | 0.002 |
| Parents status (separated vs living together*) | 5.153 | <0.001 | 3.887 | 6.419 | 0.04 |
| House crowding index | -0.475 | 0.204 | -1.207 | 0.257 | 0.001 |
| Physical activity score | -0.004 | 0.801 | -1.659 | -0.932 | 0.001 |
| Mount Lebanon (compared to Beirut) | 2.574 | 0.001 | 1.070 | 4.079 | 0.007 |
| North Lebanon (compared to Beirut) | 0.569 | 0.445 | -0.893 | 2.031 | 0.001 |
| South Lebanon (compared to Beirut) | -0.879 | 0.258 | -2.402 | 0.645 | 0.001 |
| Bekaa (compared to Beirut) | -0.820 | 0.260 | -2.248 | 0.608 | 0.001 |
| Note: In the global model, the independent variable is Parents status (living together* vs. separate). Covariates are: age, gender, mouhafaza, house crowding index and physical activity score. | | | | | |
| *Reference group | | | | | |
